# A mixed-methods study on toilet hygiene practices among Chinese in Hong Kong

**DOI:** 10.1186/s12889-019-8014-4

**Published:** 2019-12-10

**Authors:** Dan Wu, Tai Pong Lam, Hoi Yan Chan, Kwok Fai Lam, Xu Dong Zhou, Jia Yao Xu, Kai Sing Sun, Pak Leung Ho

**Affiliations:** 1 Department of Family Medicine and Primary Care, The University of Hong Kong, Hong Kong, China; 2 Faculty of Infectious and Tropical Diseases, London School of Hygiene and Tropical Medicine, London, UK; 3 Department of Statistics and Actuarial Science, The University of Hong Kong, Hong Kong, China; 4 School of Public Health, Zhejiang University, Hangzhou, China; 5 Department of Microbiology, and Carol Yu Center for Infection, The University of Hong Kong, Hong Kong, China

**Keywords:** Public toilet, Hygiene practices, Infectious diseases, China

## Abstract

**Background:**

Public toilets are a common transmission vector of infectious diseases due to environmental contamination. Research on Chinese people’s hygiene practices in public lavatories are lacking. This study examined Chinese people’s hygiene practices in public lavatories in Hong Kong.

**Methods:**

We conducted qualitative interviews and a self-administered questionnaire survey with local residents from June 2016 to April 2018. Four focus group discussions and three individual interviews informed the design of the questionnaire. We recruited interviewees and survey respondents via social service centers. The interviews and questionnaire focused on the public’s daily practices and hygiene behaviors in public toilets. Content analysis of qualitative data was conducted. Multivariable logistic regressions were used to examine the association between age and toilet hygiene behaviors.

**Results:**

Our qualitative component revealed a range of handwashing practices, from not washing at all, washing without soap, to washing for a longer time than instructions. Other toilet use practices were identified, such as not covering toilet lid before flushing and stepping on toilet seats due to dirtiness, and spitting into toilet bowls or hand basin.

Totally, 300 respondents completed the questionnaire. Among them, 212 (70.9%) were female and 246 (86.1%) were aged 65 or below. More than two thirds always washed hands with soap (68.7%) and dried hands with paper towels (68.4%). Up to 16.2% reported stepping on toilet seats and 43.9% never covered the toilet lid before flushing. Over one fourth (26.4%) spit into squat toilets/ toilet bowl. Regression analyses showed that the elderly group were less likely to report stepping on toilet seats (adjusted odds ratio, AOR = 0.17, 95%CI 0.03–0.88), flushing with the toilet lid closed (AOR = 0.40, 0.16–0.96), but more likely to spit into squat toilets/ toilet bowl (AOR = 4.20, 1.50–11.74).

**Conclusions:**

Hong Kong Chinese’s compliance to hygiene practices in public toilets is suboptimal. Stepping on toilet seat is a unique Chinese practice due to the dirtiness of toilet seats. Spitting practices may increase the risk of airborne infectious diseases and need improvement. Measures are needed to improve toilet hygiene behaviors, including public education campaigns and keeping toilet environment clean.

## Background

In public health perspectives, hygiene measures are most effective to control the outbreak of novel infectious diseases [1, 2], as well as to reduce the incidence of common respiratory, diarrheal and gastrointestinal illnesses [3]. Personal hygiene measures are applied to control transmission of bacterial and viral infections in the community [2].

Regarding use of public toilets, common hygiene measures include hand washing, drying hands, disinfecting or covering the toilet seat surface before use, and flushing the toilet with the lid closed [4–6]. Studies have shown that skin, gut and vaginal micro-organisms are prevalent on public toilet surfaces [7]. Infection risks of microorganisms in public toilets are mainly related to infective doses of pathogens. Probability of transmission is high for certain enteric pathogens such as norovirus and enterohemorrhagic *E. coli* (EHEC) in which < 100 cells are sufficient for infection [8]. It has been shown that disinfection of seats with wipes resulted in a 50-fold reduction in mean bacterial counts [9, 10]. Potentially infectious aerosols may be produced in large quantities during flushing [11, 12]. Toilet plume is a contributor to the transmission of enteric diseases [5]. Despite the above studies, there has been limited research on various inappropriate behaviors such as spitting into the urinal or toilet bowls and stepping on the toilet seat. Spitting in the urinal is an interesting phenomenon and of special concern as it may increase risk of transferring respiratory diseases when the urine splashes back into the air [13].

Various factors can affect actual hygiene practices. Studies have shown that individual knowledge and attitudes can influence their practices [2, 14, 15]. Demographic factors are also associated with the public’s practices of hygiene measures. Females were found to be more likely than males to wash their hands after using the toilet [16–19]. Besides, other influencing factors included family influence, education, peer influence and work experiences [20].

Previous qualitative studies explored the attitudes of the public towards hygiene behaviors. Western studies have found some of the public dismissed the hygiene behaviors as they considered them bothersome and inconvenient. Some also thought that infection was difficult to be controlled and one had to “accept fate”. And yet, some had a belief that the infection would happen to others instead of themselves [21, 22]. Some psychological studies also found that some people might spit after seeing dirty toilet stuff [23, 24]. However, little is known about hygiene behaviors in public toilets among Chinese.

Our study aimed to explore toilet hygiene behaviors of the Hong Kong Chinese by using a mixed-methods approach, including qualitative interviews and a questionnaire survey. The findings can enhance the understanding of professionals in fields of infection control, health education and health policy on the toilet hygiene practice.

## Material and methods

### Qualitative approach

Focus groups and individual interviews were conducted from June 2016 to January 2017 to explore in-depth views of the general public on the study topic. Focus group interviews can establish quickly a range of perspectives on a topic as the group dynamics and interactions facilitate generating rich information, while in individual interviews, participants can freely share information without any concerns that may arise in front a group. We purposively recruited adult participants (aged 18 or over) with a wide range of demographic characteristics (e.g. age, sex and income) and experience in the sample. We sent invitation letters with reply slips to social services centers in different districts of Hong Kong asking for their help to recruit participants. Potential participants who agreed to participate in the interviews were contacted by telephone. Four focus group interviews were held, of which two groups were for males and two for females. Three additional individual interviews were conducted to supplement the focus groups to see if more detailed experiences could help us to generate new themes. Two men and a woman from young and middle age groups were recruited and interviewed individually. The recruitment process ceased at the point of data saturation at which repetitive findings were seen. HKD100 (US$12.8) was offered to each interview subjects as an incentive for participation.

Each focus group interview lasted over 1 hour, while individual interviews lasted about 45 min. We used the same topic guide which was developed by the research team for both focus group discussions and individual interviews (Supplementary file 1). The interview questions focused on participants’ views and hygiene practices in public toilets, enablers and barriers to complying with recommended measures. The interviews were conducted in Cantonese (the local dialect) and audio-recorded, which were then transcribed verbatim. The accuracy of the transcripts was checked against the audio recordings. Using the content analysis approach described by Hsieh and Shannon [25], coding categories were inductively derived from the text data and a codebook was generated. Then we used the codebook to guide the analysis of the rest of the interviews and the data were coded independently by two investigators. The coding consistency between the two sets was checked and the majority of the codes were consistent. Inconsistencies were resolved by discussion between the two investigators to reach an agreement for a common theme. The key themes identified from the qualitative findings were incorporated in the design of a questionnaire for the quantitative survey.

### Quantitative approach

A questionnaire was developed based on published evidence [26, 27] as well as the themes identified from the focus group and individual interviews. The questionnaire asked about knowledge, attitudes, practices, enablers, barriers, expectations on toilet hygiene policies, and demographic information (Supplementary file 2). The questionnaire was pilot-tested for its face- and content-validity with 10 laymen. All subjects rated most of the items as comprehensible and relevant. Minor modifications were made based on the feedback.

A cross-sectional survey was conducted among the general public between July 2017 and April 2018. The target population were Chinese aged 18 or above residing in Hong Kong. We sent invitation letters to 45 community centers that served young adults / family / elderly in all 18 districts in HK, and 10 community centers accepted the invitation. Social workers at the community centers helped us recruit participants. Around 20 to 40 questionnaires were distributed at each center to participants who were visiting the center on the surveying days. Most participants completed the questionnaire by themselves. For some elderly respondents who had difficulties reading, our research assistants were on site to support and assist their reading and understanding of the questionnaire. An incentive of HKD 20 (US$2.6) was provided as a token of gratitude. Subjects who had intellectual disability or were not able to communicate in Chinese were excluded.

The data were analyzed using SPSS (Version 25). Descriptive analysis was conducted. Hygiene behavioral variables were recoded. The responses “sometimes” and “always” were grouped for further analyses due to small cell numbers of some variables. Multivariable logistic regression analyses were carried out to examine the association between age and hygiene behaviors, controlling for gender, education, marriage and birthplace. A *p*-value < 0.05 was considered statistically significant.

## Results

### Participants

We recruited 25 participants in 4 focus groups (6–7 per group), consisting of 10 males and 15 females. Their ages ranged from 18 to 86 years. Slightly more than one fifth (20.8%) of them received tertiary education, 37.5% received secondary education, and 41.7% received primary education. In addition, we conducted individual interviews with three participants who were aged from 22 to 56 years. For the questionnaire survey, we recruited a total of 300 participants from 10 community centers across Hong Kong.

### Qualitative findings

#### Handwashing

Participants were asked about whether they washed hands after using toilet. We identified a spectrum of hand washing behaviors – not washing at all, washing without soap, washing with soap but not according to instructions, washing with soap according to instructions, over-washing for a longer time than instructions. Some personal habits or conditions appeared to play a dominant role in driving handwashing rather than scientific reasons.

- *P2: To minimize the amount of bacteria on my own hands.*
- *P5: Sometimes it’s because I’m afraid of the smell.*
- *P4: I would feel better after washing my hands.*

*(Focus group 3 - young to middle aged females)*

*If you touch your own urine during urination, then you would wash [your hands]. If there is none, there is no need to wash [your hands]. (Focus group 4 – male elders, P2).*

Some perceived no difference between washing hands with and without soap after urination.

*I wash my hands with water only after urination. I rarely use soap. (Focus group 4 – male elders, P2).*

Some used soap for the purpose of keeping clean and a better feeling about smell.

*It is cleaner and feels refreshing if washing hands with soap. (Focus group 4 – male elders, P3).*

Some participants disagreed with the necessity of complying to guideline of handwashing and considered the procedures as “too complicated” (Individual interview 3, middle aged male). Using soap and rubbing hands would also inevitably prolong their washing time, creating reluctance to following guidelines (Focus group 4 – male elders, P2). Some were just “not used to using soap” and had “no such habit” (focus group 1, housewives). On the other hand, some participants were concerned about being “over-clean”, or were “obsessed with handwashing” *(focus group 1 - housewives).*

Additionally, participants mentioned that soap was not always available in public toilets. Even provided, the soap might be very watery. They suspected that water was added to dilute the soap. Some others wanted to reduce their contact with the utilities when they found the toilet environment dirty and were reluctant to use soap if they had to press the soap button.

*I want to have less contact with the soap button which has been pressed by so many people. The button is contaminated. I will use the soap if it’s automatic but not when it requires manual pressing. (focus group 2, male youths).*

#### Public toilet use behaviors

Some participants would cover the toilet lid at home but not in public toilets when they flushed because some covers looked too dirty to touch.

*In public toilets the covers are too dirty that I would not even touch them. Therefore, there are times I don’t cover it.* (*Individual interview, female, aged 42, tertiary educated).*

Some were worried that the toilet lid might be soiled by the feces, which “splashed onto the cover when you flushed with the cover closed” (Focus group 3 - young to middle aged females). Some participants stepped on the toilet seats to avoid direct contact as they perceived the toilets unclean.

*I want to avoid and minimize contact with the toilet, because it feels unsafe if I had contact with it. (Interviewer: It’s quite difficult to step on it) Yeah, it is a bit difficult, but I have no choice. I feel that it would be cleaner if I do it this way. (Individual interview 3, middle aged male)*

Our interviews also revealed that participants spitted into urinals, toilet bowls, or hand basin.

*P2: If you need to spit during urination, then of course [you would spit] into the urinal. If you are washing your hands, then of course [spitting] into the hand basin.*

*(Focus group 4 – male elders)*

*Because there is a pool of water in the toilet bowl. The sputum will sink to the bottom and would not remain on the surface or suspend in the air. (Individual interview 3, middle aged male).*

### Survey finding

Table 1 shows the demographic backgrounds of the respondents. Among 300 respondents, 212 (70.9%) were female and 246 (86.1%) were aged below 65 years. Forty-four (14.8%) received primary education or less, and 104 (36.5%) were in the low-income group. Over half of them (58.0%) were married.

**Table 1:** Demographic characteristics of survey participants (*N* = 300)

	n	%
Gender		
M	87	29.1
F	212	70.9
Age		
< 40	128	44.8
40–65	118	41.3
≥ 65	40	14.0
Education		
Primary or below	44	14.8
Secondary	163	54.9
Tertiary	90	30.3
Household Income		
Low	104	36.5
Middle	147	51.6
High	34	11.9
Marital status		
Married	171	58.0
Unmarried	124	42.0

Note: column cell numbers do not add up to the total sample size due to missing values

#### Public toilet using behaviors

Table 2 shows the participants’ hygiene behaviors of using public toilets. The majority reported hand hygiene behaviors, such as always cleaning the toilet seat with tissue paper (48.1%) or alcohol (22.5%), washing hands with soap (68.7%) and drying hands with paper towels (68.4%). Nearly half (45.6%) of them did not sit on the public toilet seat but 16.2% reported stepping on it. While 98.3% of the participants reported that they flushed after using the toilet, 43.9% of them did not flush with the toilet lid closed. Over a quarter (26.4%) spit into squat toilets / toilet bowl.

**Table 2:** Public toilet use and hygiene behaviors of the respondents, n (%) (N = 300)

	Never	Sometimes	Always
Clean the toilet seat with alcohol	123 (43.3)	97 (34.2)	64 (22.5)
Clean the toilet seat with tissue paper	54 (18.6)	97 (33.3)	140 (48.1)
Put tissue paper on the toilet seat before using	103 (35.6)	100 (34.6)	86 (29.8)
Sit on the toilet seat	130 (45.6)	77 (27.0)	78 (27.4)
Step on the toilet seat	237 (83.7)	36 (12.7)	10 (3.5)
Flush after using the toilet	5 (1.7)	10 (3.4)	277 (94.9)
Flush with the toilet lid closed	127 (43.9)	104 (36.0)	58 (20.1)
Wash your hands with water	3 (1.0)	5 (1.7)	283 (97.3)
Wash your hands with soap	11 (3.8)	80 (27.5)	200 (68.7)
Dry your hands with paper towels	18 (6.1)	75 (25.5)	201 (68.4)
Dry your hands with a hand dryer	68 (23.1)	172 (58.5)	54 (18.4)
Spit into urinals^a^ (*n* = 85)	54 (63.5)	28 (32.9)	3 (3.5)
Spit into squat toilets/ toilet bowl	211 (73.5)	69 (24.0)	7 (2.4)

^a^For male participants only

Note: row cell numbers do not add up to the total sample size due to missing values

#### Views towards toilet use behaviors with increased risk for infections

Table 3 demonstrates participants’ views towards toilet use behaviors with increased risk for infections. Up to 31.8% of survey participants regarded frequent use of public toilets as a risk behavior for infections. Over 75% agreed that not washing hands after toilet, not flushing the toilet, and touching contaminated toilet facilities might increase risk of infection. However, only 53.4% of the participants felt that not using soap to wash hands after toilet would increase risk. A total of 58.7% of participants felt that spitting into hand basins would increase risk, and nearly one third of male participants perceived increased risk of infection was associated with spitting into urinals. Almost half (48.3%) perceived sitting on the toilet seat would increase risk for infections. About one-third (34.2%) agreed that flushing the toilet without the lid closed was a risk factor.

**Table 3:** Views towards toilet use behaviors with increased risk for infections, n (%) (*N* = 300)

	Yes
Not washing one’s hands after toilet	233 (78.2)
Not flushing the toilet	229 (76.8)
Touching contaminated toilet facilities	229 (76.8)
Spitting into hand basins	175 (58.7)
Not using soap to wash one’s hands after toilet	159 (53.4)
Sitting on the toilet seat	144 (48.3)
Spitting into urinals^a^	28 (32.2)
Flushing the toilet without the lid closed	102 (34.2)
Frequent use of public toilets	94 (31.8)
Not drying one’s hands after washing them	43 (14.4)

^a^Among male participants

#### Association between age and public toilets using behaviors

Association between age and hygienic behaviors of using public toilets is shown in Table 4. Compared to participants who were younger than 40, respondents in the middle-aged (adjusted odds ratio or AOR = 0.51, 95% CI 0.28–0.94) and elderly (AOR = 0.16, 0.06–0.45) groups were significantly less likely to clean the toilet seat with alcohol. Respondents in the elderly group were less likely to report stepping on toilet seats (AOR = 0.17, 0.03–0.88), flushing with the toilet lid closed (AOR = 0.40, 0.16–0.96), but more likely to spit into squat toilets/ toilet bowl (AOR = 4.20, 1.50–11.74). Other behaviors were not significantly associated with age.

**Table 4:** Association between age and public toilet use behaviors

Behavioral variables ^a^	Model 1	Model 2
	Crude Odds Ratio (95%CI)	Adjusted Odds Ratio (95%CI)
Clean the toilet seat with alcohol		
< 40	Ref	Ref
40–65	0.71 (0.42–1.19)	0.51 (0.28–0.94)*
65 or above	0.21 (0.09–0.49)***	0.16 (0.06–0.45)**
Sit on the toilet seat		
< 40	Ref	Ref
40–65	0.71 (0.42–1.18)	0.77 (0.43–1.39)
65 or above	2.60 (1.10–6.18)*	2.65 (0.93–7.50)
Step on the toilet seat		
< 40	Ref	Ref
40–65	0.75 (0.39–1.46)	0.49 (0.22–1.06)
65 or above	0.40 (0.11–1.42)	0.17 (0.03–0.88)*
Flush with the toilet lid closed		
< 40	Ref	Ref
40–65	0.69 (0.41–1.15)	0.64 (0.36–1.14)
65 or above	0.45 (0.21–0.95)*	0.40 (0.16–0.96)*
Dry your hands with a hand dryer		
< 40	Ref	Ref
40–65	0.68 (0.37–1.24)	0.64 (0.32–1.30)
65 or above	0.43 (0.19–0.97)*	0.47 (0.17–1.30)
Spit into squat toilets/ toilet bowl		
< 40	Ref	Ref
40–65	1.54 (0.85–2.79)	1.70 (0.84–3.44)
65 or above	2.75 (1.25–6.06)*	4.20 (1.50–11.74)**

^a^ responses of “sometimes” and “always” for behavioral variables were grouped for binary logistic regression analysis and reference response was “never”

Model 1: included age only in the modelling

Model 2: gender, education, marriage and birthplace were controlled in the modelling

^*^: < 0.05; ^**^: < 0.01; ^***^: < 0.001

#### Spitting into toilets

Table 5 shows people’s perception towards spitting into urinals. 43% of our survey respondents agreed that the urinal would be the most convenient place to spit, and there were no other preferable places to do so. However, 70% did not think that the urinal was a sanitary place to spit. Remarkably, almost 40% of our respondents believed that most men had the habit of spitting in the urinal before urinating. Only a few attributed the behavior to the dirty environment of public toilet or stress release.

**Table 5:** Practices and reasons for spitting into toilets, n (%) (N = 300)

Do you think that	Strongly disagree	Disagree	Agree	Strongly Agree
Most men would spit in the urinal before urinating	38 (13.2)	136 (47.2)	105 (36.5)	9 (3.1)
The environment/smell of public toilet makes users want to spit	62 (20.8)	133 (44.6)	88 (29.5)	15 (5.0)
Spitting before urinating helps to release pressure	95 (32.1)	183 (61.8)	16 (5.4)	2 (0.7)
Spitting in the urinals because there is no other preferable place to spit	58 (19.7)	105 (35.6)	119 (40.3)	13 (4.4)
It is most convenient to spitting in urinals in public toilets	51 (17.3)	118 (40.0)	113 (38.3)	13 (4.4)
It is most hygienic to spitting in urinals in public toilets	81 (27.4)	138 (46.6)	68 (23.0)	9 (3.0)

Note: row cells do not add up to the total sample size due to missing values

## Discussion

Good personal hygiene is essential in preventing infection. Dirty public toilets are known to be a source of bacteria and viruses [5, 7, 8, 12]. However, little is known about people’s hygiene practices in their daily use of public toilets. Our study extends the literature by exploring hygiene behaviors in public toilets among Chinese in Hong Kong. Age was a potential correlate with some public toilet hygiene behaviors.

We found that slightly more than two thirds had adequate hand hygiene habits who always washed hands with soap and dried hands with paper towels after washing. These are similar to a previous local study during the H1N1 pandemics [19]. The compliance of 60–70% are yet suboptimal when compared to the hand hygiene study findings of other countries such as Uganda and Turkey [4, 28]. Handwashing and hand drying have long been recognized as the most important measure in reducing transfer of bacteria to other surfaces and thus the risk of bacterial infection [29–31]. Our qualitative interviews also indicated that people do not, however, properly comply with hand hygiene measures because that would take more time to execute and maintain the practice. Some steps of the practice were often neglected, and people tended to overlook the long-term impacts of poor handwashing habits on one and others’ health. While there have been public health promotions such as Dos and Don’ts illustrations and slogans in the community, more campaigns might be needed to aid the understanding of the reasons for the standard procedures in improving hand hygiene.

Our study showed many flushed toilets without having the toilet lid closed. Previous research suggested closing the toilet lid before flushing can avoid toilet plume and reduce the chance of spreading aerosols that may carry gastrointestinal pathogens such as *Clostridium difficile* and norovirus [5, 8, 32]. Our participants did not put down the toilet lid mostly because they felt public toilet lids were too dirty to touch. The refurbishment and cleanliness of the facilities appear paramount to hygiene compliance. While the Hong Kong government noticed and addressed such needs [33, 34], other tailored measures are worth consideration. More specifically, increasing the frequency of cleaning public toilets, providing clean materials such as alcohol disinfection liquids, and innovative technology such as automatic closure may be good starting points [34–36]. In an attempt to reduce the transmission of multidrug-resistant organisms such as methicillin-resistant *Staphylococcus aureus* and vancomycin-resistant enterococci, all public hospitals in Hong Kong have implemented a policy to request patients to use alcohol wipes to clean the toilet seats before use [10, 37, 38].

We noted that many spit into urinals or toilet bowls. Moreover, the elderly group were more likely to do so. Spitting is a known factor that may increase the spread of airborne infections, such as tuberculosis [39, 40]. Besides tuberculosis, spitting and flushing without closing the toilet lids may generate droplets and aerosols containing human enteric viruses that is well known for its remarkable ability to survive in the environment [41]. Possible explanations included that people held misconceptions about spitting into urinals/toilet bowls, such as spitting in toilets was most hygienic, and poor toilet environment might also stimulate men’s impulsion to spit. Another reason was that many found it most convenient to spit into the toilet or there was no better place to do so. Public education about how to properly dispose sputum, especially for elderly users, and cleaning the toilet environment may be worth consideration. In addition, provision of sufficient tissue paper and rubbish bin might reinforce more hygienic disposal.

Lastly, nearly half never sat on public toilet and many reported stepping on toilet seats before. Our interviews revealed the main reason for doing so was the dirtiness of public toilets. Stepping on toilet makes the toilet dirtier, and more importantly, it is also unsafe for people. There were news reports about getting hurt badly by a broken toilet as people were stepping on it [42, 43]. Providing disposal toilet covers or disinfection wipes in public toilets may help facilitate safer toilet use practices. Given the fact that the majority did not actually sit on public toilet, building more squatting toilet in public facilities may also be a worthwhile strategy to prevent people getting injured from the practice.

The study has several limitations. First, we recruited respondents from those who were visiting social service centers. This might lead to selection bias as certain demographic subgroups may be more likely to utilize community services. Our study findings, however, provided a snapshot of how general public used public toilets, and tapped into a neglected public health issue in Hong Kong. Second, we conducted the study in a developed city in China. Our study findings may have limited representativeness of the whole Chinese population. But they may reflect to some extent the situation in developed areas in the country. Third, our study used a self-administered questionnaire in which respondents may give socially desirable responses. Non-hygienic toilet use behaviors might be underestimated.

## Conclusions

While common toilet hygiene measures have long been promoted among the public in Hong Kong, our findings indicated that the local Chinese’s compliance was only suboptimal, and the hygiene measures were far from fully practiced. Some steps of these practices could be easily overlooked and neglected due to the time- and resources-consuming process. The dirtiness and poor-maintenance of the facilities in local public toilets appeared to be the predominant barrier to toilet hygiene compliance. We recommend increasing the frequency of cleaning public toilets and providing sufficient toilet paper and cleaning agents, in order to improve and reinforce the quality of toilet hygiene behaviors.

## Supplementary information

**Additional file 1.** Survey questionnaire.

**Additional file 2.** Focus group and individual interview guideline

## Data Availability

The datasets used and/or analysed during the current study are available from the corresponding author on reasonable request.

## Abbreviations

AOR: Adjusted odds ratio
EHEC: Enterohemorrhagic *E. coli*
