# Supplementary material for: A mixed-methods study on toilet hygiene practices among Chinese in Hong Kong
Source: BMC Public Health. 2019 Dec 10;19:1654. doi: 10.1186/s12889-019-8014-4 (PMC6902477; doi:10.1186/s12889-019-8014-4)
Supplement: Supplementary file 1 — Additional file 1. Survey questionnaire. [file 12889_2019_8014_MOESM1_ESM.doc]

**Toilet Hygiene Practices Survey among Hong Kong Chinese**

**Organized by Department of Family Medicine and Primary Care,**

**The University of Hong Kong**

We would like to understand your opinions on public toilet facilities and your personal hygiene measures.

***Please 🗸 the appropriate box.***

| **1.** | **When you use the toilet, you** | **Never** | **Sometimes** | **Always** |
| --- | --- | --- | --- | --- |
| a | Clean the toilet seat with alcohol | □ | □ | □ |
| b | Clean the toilet seat with tissue paper | □ | □ | □ |
| c | Put tissue paper on the toilet seat before using | □ | □ | □ |
| d | Sit on the toilet seat | □ | □ | □ |
| e | Step on the toilet seat | □ | □ | □ |
| f | Flush after using the toilet | □ | □ | □ |
| g | Flush with the toilet lid closed | □ | □ | □ |
| h | Wash your hands with water | □ | □ | □ |
| i | Wash your hands with soap | □ | □ | □ |
| j | Dry your hands with paper towels | □ | □ | □ |
| k | Dry your hands with a hand dryer | □ | □ | □ |
| l | Spit into urinals | □ | □ | □ |
| m | Spit into squat toilets/ toilet bowl | □ | □ | □ |

***2. Which of the following behaviours put health at risk? (You may choose more than one option)***

| □ Sitting on the toilet seat | □ Not drying one’s hands after washing them | □ Frequent use of public toilets |
| --- | --- | --- |
| □ Not washing one’s hands after toilet | □ Flushing the toilet without the lid closed | □ Spitting into urinals |
| □ Not using soap to wash one’s hands after toilet | □ Not flushing the toilet | □ Spitting into hand basins |
| □ Touching contaminated toilet facilities | | |

***3. Practices and reasons for spitting into toilets***

|  | Do you think that... | Strongly disagree | Disagree | Agree | Strongly Agree |
| --- | --- | --- | --- | --- | --- |
| a | Most men would spit in the urinal before urinating | □ | □ | □ | □ |
| b | The environment/smell of public toilet makes users want to spit | □ | □ | □ | □ |
| c | Spitting before urinating helps to release pressure | □ | □ | □ | □ |
| d | Spitting in the urinals because there is no other preferable place to spit | □ | □ | □ | □ |
| e | It is most convenient to spitting in urinals in public toilets | □ | □ | □ | □ |
| f | It is most hygienic to spitting in urinals in public toilets | □ | □ | □ | □ |

**We would like to collect some of your personal information for research purpose. All information of yours will remain confidential.**

| 4. | Gender: □ Male □ Female |
| --- | --- |
|  |  |
| 5. | Marital Status   | □ Single | □ Married | □ Living together | □ Divorced | □ Widowed | | --- | --- | --- | --- | --- | |
|  |  |
| 6. | Age： ________________________ |
|  |  |
| 7. | Education level   | □ No schooling/kindergarten | □ Primary | □ F.1-F.3 Lower secondary | | --- | --- | --- | | □ F.4-F.7 Upper secondary | □Tertiary:non-degree | □ Tertiary: Degree | |
|  |  |
| 8. | Monthly household income (in Hong Kong Dollars)   | □ 5000 or below | □ 5001-10000 | □ 10001-25000 | | --- | --- | --- | | □ 25001-40000 | □ 40001-60000 | □ 60001or above | |
