# Supplementary material for: A mixed-methods study on toilet hygiene practices among Chinese in Hong Kong
Source: BMC Public Health. 2019 Dec 10;19:1654. doi: 10.1186/s12889-019-8014-4 (PMC6902477; doi:10.1186/s12889-019-8014-4)
Supplement: Supplementary file 2 — Additional file 2. Focus group and individual interview guideline [file 12889_2019_8014_MOESM2_ESM.docx]

**Focus group and individual interview guideline**

What do you think about the environment of public toilets that you go most often? What hygienic concerns do you have?

What hygiene measures (including lay practice) did you adopt for public toilets and household toilets? Why?

What do you think about following behaviours? Would you share your views?

- not washing hands
- not washing hands with soap
- not drying hands after washing
- spitting in the urinal or toilet
- standing far from the urinal *[for males only]*
- not disinfecting the toilet seat surface before use
- stepping on the toilet seat
- flushing the toilet without the lid closed
- not pushing the flush button

What channels do you get to know information on toilet hygiene measures? What are these measures about? Do you understand the relationship between these behaviors and potential infections?

Which measure(s) have you adopted? Why or why not? What may facilitate you to comply with these measures? What may prevent you from using these measures?

Do you persuade/educate your family members (e.g. children) to use similar measures?

What hygiene measure polices you expect the health authorities to implement in public toilets?
